# Neuropsychiatric symptoms in epileptic seizures. A case report

**DOI:** 10.1192/j.eurpsy.2025.1456

**Published:** 2025-08-26

**Authors:** B. Lopez Abellan, P. Abril Bohórquez, N. Linero Rios, R. Justicia González, N. Echeverría Hernández

**Affiliations:** 1 Psiquiatría, Complejo Asistencial de Ávila, Ávila, Spain

## Abstract

**Introduction:**

Epilepsy is the most common chronic neurological disease in the general population. The role of the psychiatrist in this pathology is to consider the epileptic diagnosis within the diagnostic possibilities, its psychosocial consequences and the psychological and cognitive effects of common antiepileptics. It has been reported that 30% to 50% of all individuals with epilepsy present, at some point in their lives, psychiatric difficulties. An epileptic seizure is a transient paroxysmal pathophysiological alteration of brain function caused by spontaneous excessive neuronal discharge. Symptoms will depend fundamentally on the cerebral origin of the seizure and the spread of activity in the brain. Finally, to better understand the clinical case that will be presented, it is important to differentiate between partial epileptic seizures, which involve epileptiform activity in localized brain regions, and generalized seizures, which affect the entire brain.

**Objectives:**

The main objective of this work is to review the current scientific evidence on psychiatric symptons in epilepsy.

**Methods:**

The case of a 75-year-old man with a neurological history and abrupt appearance of atypical psychiatric symptoms is presented. A detailed search was performed on UpToDate with the search terms “Epilepsy” and “psychiatric pathology.”

**Results:**

This is a 75-year-old man with an organic history of repeated cerebral strokes and vascular epilepsy who attended the Emergency Department due to the onset of autolytic ideation abruptly and without a biographical trigger. After the express request by psychiatry for a neurological study, an EEG was performed, where epileptiform activity was observed in the frontal lobe and a diagnosis of right frontal focal status epilepticus secondary to a chronic cerebrovascular lesion was diagnosed. Preictal events in complex partial epilepsy include autonomic sensations (gastric repletion, flushing and changes in breathing), cognitive sensations (déjà vu, jamais vu, forced thoughts), affective states (fear, panic, depression, euphoria) and automatisms.

**Conclusions:**

Status epilepticus constitutes one of the main neurological emergencies. Convulsive status epilepticus is a life-threatening situation that requires immediate pharmacological treatment and life support measures, as well as recognition and treatment of a possible triggering cause. It is a prolonged epileptic seizure or one that repeats itself in time intervals that are short enough so that the patient does not regain consciousness between episodes. Among the possible symptoms in epilepsy with a focus on the frontal lobe, atypical behavioral alterations are included that can go unnoticed and be referred to psychiatry.

In conclusion, this case shows the need for organic screening in those patients with atypical symptoms that do not fit into the major neuropsychiatric syndromes.

**Disclosure of Interest:**

None Declared

